# Role of the Molecular Environment in Quenching the Irradiation-Driven Fragmentation of Fe(CO)_5_: A Reactive Molecular Dynamics Study

**DOI:** 10.1021/acs.jpca.2c08756

**Published:** 2023-04-19

**Authors:** Benjamin Andreides, Alexey V. Verkhovtsev, Juraj Fedor, Andrey V. Solov’yov

**Affiliations:** † J. Heyrovský Institute of Physical Chemistry, Czech Academy of Sciences, Dolejškova 3, 18223 Prague, Czech Republic; ‡ MBN Research Center, Altenhöferallee 3, 60438 Frankfurt am Main, Germany

## Abstract

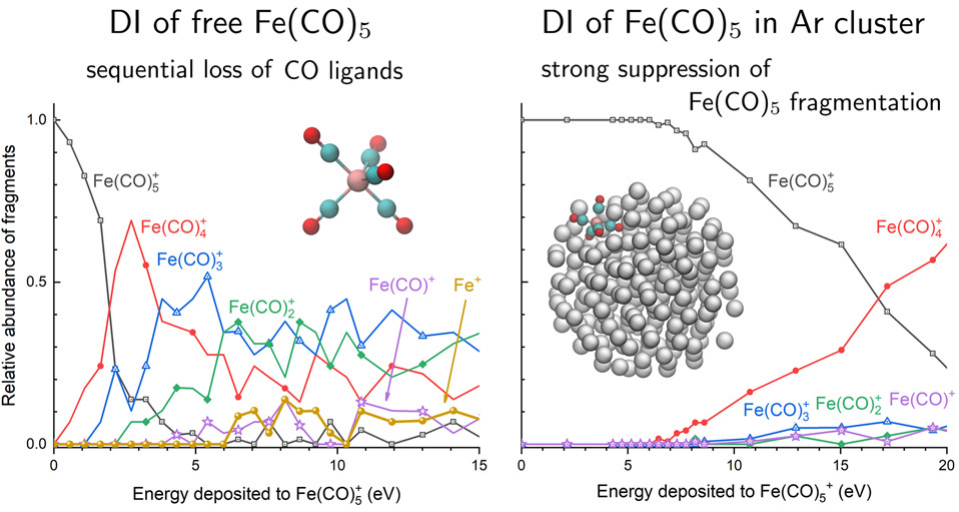

Irradiation-driven fragmentation and chemical transformations of molecular systems play a key role in nanofabrication processes where organometallic compounds break up due to the irradiation with focused particle beams. In this study, reactive molecular dynamics simulations have been performed to analyze the role of the molecular environment on the irradiation-induced fragmentation of molecular systems. As a case study, we consider the dissociative ionization of iron pentacarbonyl, Fe(CO)_5_, a widely used precursor molecule for focused electron beam-induced deposition. In connection to recent experiments, the irradiation-induced fragmentation dynamics of an isolated Fe(CO)_5_^+^ molecule is studied and compared with that of Fe(CO)_5_^+^ embedded into an argon cluster. The appearance energies of different fragments of isolated Fe(CO)_5_^+^ agree with the recent experimental data. For Fe(CO)_5_^+^ embedded into an argon cluster, the simulations reproduce the experimentally observed suppression of Fe(CO)_5_^+^ fragmentation and provide an atomistic-level understanding of this effect. Understanding irradiation-driven fragmentation patterns for molecular systems in environments facilitates the advancement of atomistic models of irradiation-induced chemistry processes involving complex molecular systems.

## 1 Introduction

Irradiation-driven chemistry (IDC) processes induced by the interaction of different types of radiation (X-rays, electrons, and ion beams) with molecular systems are exploited in many modern and emerging technologies. For instance, IDC processes play an important role in ion-beam radiotherapy^1,2^ that exploits the ability of charged heavy particles to inactivate living cells due to the induction of complex DNA damage.^3−5^ IDC transformations in molecular films have been studied in relation to astrochemistry.^6,7^ Such transformations occur during the formation of cosmic ices in the interstellar medium due to the interplay of molecular adsorption on a surface and surface irradiation.^8^

Electron irradiation-induced chemistry of organometallic molecules is central to focused electron beam-induced deposition (FEBID)—a technology for the controllable fabrication of complex nanostructures with nanometer resolution.^9−12^ In the FEBID process, electron-induced molecular fragmentation occurs via the dissociative ionization (DI), dissociative electron attachment (DEA), or neutral dissociation (ND) mechanisms, leading to the production of cationic, anionic, or neutral fragments, respectively.^13^ Electron-induced decomposition of adsorbed precursor molecules releases organic ligands, resulting in the clusterization of the precursor’s metallic component on a surface. The fundamental physicochemical phenomena that govern the formation, growth, and composition of deposits grown by FEBID still need to be fully understood and are the subject of ongoing research.^14−16^ Achieving this goal requires a concerted approach linking fundamental knowledge of electron-driven chemistry in FEBID^17^ with rational design and synthesis of novel precursor molecules.^18^

In recent years, much effort has been put into entangling the elementary processes, which lead to electron-induced cleavage of metal–ligand bonds; see review papers^13,14,19^ and references therein. However, the vast majority of data on the electron irradiation-induced processes involving FEBID precursor molecules is experimental. Typically, in experiments, a well-defined precursor target is crossed with a monochromatized electron beam, and the yields of reaction products are measured as a function of the projectile electron energy. Such experiments have been performed for precursor molecules in the gas phase,^20−22^ those embedded in a cluster environment,^23−26^ and those condensed on a surface as thin molecular films;^27,28^ see also the recent reviews.^14,19^

A detailed atomistic-level understanding of IDC processes (i.e., bond cleavage and further reactivity) in molecular systems can be developed through computational modeling. A rigorous quantum-mechanical description of these processes, e.g., within time-dependent density functional theory (TDDFT), is feasible for relatively small molecular systems containing, at most, a few hundred atoms and evolving on the subpicosecond time scale.^29−31^

Radiation- and collision-induced fragmentation of molecular systems on much larger time scales (from tens of picoseconds up to hundreds of nanoseconds) has been successfully studied by means of classical reactive molecular dynamics (MD)^32^ and irradiation-driven MD (IDMD)^33^ methodologies using the advanced software package MBN Explorer.^34^ These computational methodologies enable to embed random, fast, and local quantum transformations occurring in molecular systems due to chemical reactions or irradiation-induced quantum processes (e.g., bond breakage via DI or DEA) into the classical MD framework. This provides possibilities for simulations of chemical and irradiation-driven transformations of various molecular, biomolecular, and nano systems on the temporal and spatial scales inaccessible for simulations based on the *ab initio* quantum methods.^15,16,33,35−37^ Major dissociative transformations of irradiated molecular systems (such as molecular topology changes, redistribution of atomic partial charges, or alteration of interatomic interactions) are simulated by means of MD with reactive force fields, particularly the reactive CHARMM (rCHARMM) force field^32^ implemented in MBN Explorer.

In this study, reactive MD simulations are performed to analyze the effects of the molecular environment on the fragmentation of molecular systems after their ionization. As a case study, we consider electron-impact-induced DI of iron pentacarbonyl, Fe(CO)_5_, one of the most common FEBID precursors for the fabrication of iron-based nanostructures.^38−41^ The metal–ligand separation and the CO ligand dissociation processes are simulated using the reactive rCHARMM force field^32^ and quantified by analyzing appearance energies for different molecular fragments. The role of the molecular environment is analyzed by comparing the irradiation-induced fragmentation dynamics of an isolated Fe(CO)_5_^+^ ion with that of a Fe(CO)_5_^+^ ion embedded into an argon cluster.

Two recent experimental studies provide a direct motivation for this work. Lacko et al.^20^ studied electron-impact-induced DI of Fe(CO)_5_ molecules in the gas phase. Different fragment ions corresponding to a sequential loss of individual CO ligands (down to a bare Fe^+^ fragment) were observed, and the appearance energies of these ions were determined with high resolution. Lengyel et al.^24^ studied the DI of Fe(CO)_5_ picked up on argon clusters with a mean size of several hundred argon atoms. Strong suppression of ligand dissociation and a change in the Fe(CO)_5_ ionization mechanism were observed.^24^ The simulation results reported in this study agree with the results of these gas-phase and cluster-beam experiments. For isolated Fe(CO)_5_^+^, the main benchmark of the simulations—appearance energies of the individual fragment ions—is in good quantitative agreement with the experimental data. For Fe(CO)_5_^+^ embedded into an argon cluster, the simulations reproduce the experimentally observed suppression of Fe(CO)_5_^+^ fragmentation and provide an atomistic-level understanding of this effect.

The results reported in this study indicate the importance of understanding irradiation-driven fragmentation patterns for molecular systems in molecular environments. Such an understanding may facilitate the advancement of atomistic models of irradiation-induced chemistry processes involving complex molecular systems.

## 2 Computational Methodology

MD simulations of irradiation-driven fragmentation of Fe(CO)_5_^+^ have been performed by means of the MBN Explorer software package.^34^ The MBN Studio toolkit^42^ has been utilized to create the systems, prepare necessary input files, and analyze simulation outputs.

In this study, the results of MD simulations are compared with the experimental results on electron-impact-induced dissociative ionization.^20,24^ As such, a singly charged parent ion, Fe(CO)_5_^+^, is considered in the simulations. Within the utilized computational methodology based on classical reactive MD simulations, it is assumed that Fe(CO)_5_^+^ is in its ground electronic state. The electron impact ionization process studied experimentally in refs (20 and 24) can lead to electron removal from different molecular orbitals, and the cation can thus be formed in many different initial electronic states (with holes in the corresponding orbitals). Such quantum processes occurring at the initial time instant are not considered within the classical MD framework. However, it is well established that the internal conversion of excited states, e.g. via conical intersections, populates the cation’s electronic ground state.^43,44^ The conversion to the ground state is a fast process, typically proceeding within tens of femtoseconds.^45^ The energy initially stored in the electronic degrees of freedom is thus transferred to the vibrational degrees of freedom in the ground state; this represents the starting point of our simulations. The energy transferred to the vibrational degrees of freedom is taken as the excess energy in the simulations, justifying the use of classical reactive MD to characterize the fragmentation patterns.

### 2.1 Interaction Potentials

Interatomic interactions for the Fe(CO)_5_^+^ ion have been described using the reactive CHARMM (rCHARMM) force field introduced in ref (32). rCHARMM permits simulations of various molecular systems with the dynamically changing molecular topology,^46−49^ which is essential for modeling irradiation-driven transformations and chemistry. Examples of the application of rCHARMM^32^ to different molecular systems are summarized in a recent review^50^ and a book.^37^

The radial part of bonded interactions is described in rCHARMM by means of the Morse potential:
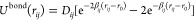
1Here *D*_*ij*_ is the dissociation energy of the bond between atoms *i* and *j*, *r*_0_ is the equilibrium bond length, and the parameter  (with *k*_*ij*_^*r*^ being the bond force constant) determines the steepness of the potential. The bonded interactions are truncated at a user-defined cutoff distance beyond which the covalent bond gets broken and the molecular topology of the system changes.

The rupture of covalent bonds in the course of simulation employs the following reactive potential for valence angles:^32^
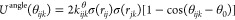
2where θ_0_ is the equilibrium angle formed by a triplet of atoms *i*, *j*, and *k*, *k*^θ^ is the angle force constant, and the function
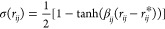
3describes the effect of bond breakage (see ref (32) for the details). The parameter *r*_ij_^*^ in eq 3 is given by
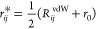
4where *r*_0_ is the equilibrium distance between two atoms involved in the angular interaction and *R*_*ij*_^vdW^ is the sum of the van der Waals radii for those atoms.

**Figure 1:**
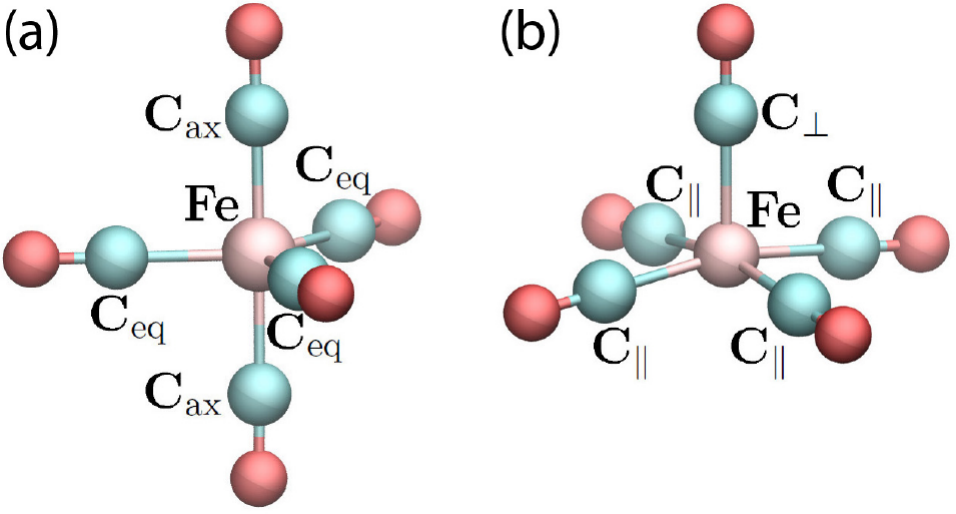
Optimized geometry of Fe(CO)_5_^+^ considered in this study: a trigonal-bipyramidal *D*_3*h*_ isomer (a) and a square-pyramidal *C*_4*v*_ isomer (b). The optimization calculations have been performed by means of MBN Explorer^34^ using the interatomic potentials given by eqs 1–5. Different atom types are indicated. The corresponding bonded and angular interactions are listed in Table 2.

Two structural isomers of Fe(CO)_5_^+^ shown in Figure 1 have been considered in this study. A trigonal bipyramidal *D*_3*h*_ isomer (panel a) corresponds to the ground-state geometry of a neutral Fe(CO)_5_ molecule in the gas phase,^51^ while a square-pyramidal *C*_4*v*_ isomer (panel b) is commonly considered as the ground-state geometry of a Fe(CO)_5_^+^ cation.^20,52,53^ In the *D*_3*h*_ symmetric structure (Figure 1a), two axial (“ax”) CO groups lie on the main symmetry axis of the ion, and three equatorial (“eq”) CO groups lie in the plane perpendicular to the main symmetry axis. In the *C*_4*v*_ symmetric structure (Figure 1b), the Fe–C_⊥_ bond is almost orthogonal to the four Fe–C_∥_ bonds, with the C_⊥_–Fe–C_∥_ angle being equal to 96.4°.

DFT-based structure optimization calculations have been performed for *D*_3*h*_ and *C*_4*v*_ structural isomers of Fe(CO)_5_ and Fe(CO)_5_^+^ by means of the Gaussian 16 software.^54^ Several combinations of exchange-correlation functionals and basis sets have been considered (see Table 1). For a positively charged Fe(CO)_5_^+^ ion, different spin states with spin multiplicities *M* = 2, 4, and 6 were considered. For the neutral Fe(CO)_5_ molecule, the triangular-bipyramidal (*D*_3*h*_) structure has the energy ∼0.09 eV lower than that of the *C*_4*v*_ isomer for all the cases considered. For the Fe(CO)_5_^+^ cation, the doublet state (*M* = 2) was found to be the lowest-energy state in most cases. The only exception is the calculations employing the M06-2X functional, which predict that the quartet state (*M* = 4) is ∼0.3–0.6 eV lower in energy than the doublet state.

**Table 1:** Ionization Energies (IE) of a Fe(CO)_5_ Molecule (in eV) Calculated at Different Levels of DFT^a^

level of theory	VIE	IE_ad_(*D*_3*h*_)	IE_ad_(*C*_4*v*_)
B3LYP/6-31+G(d)	8.62	8.04	7.80
B3LYP/Def2TZVP	8.45	7.87	7.63
CAM-B3LYP/Def2TZVP	8.33	7.68	7.44
M06-2X/6-31+G(d)	7.44	5.99	5.99
M06-2X/Def2TZVP	7.34	5.78	5.78

a VIE is the vertical ionization energy for Fe(CO)_5_. IE_ad_(*D*_3*h*_) and IE_ad_(*C*_4*v*_) are the adiabatic ionization energies for *D*_3*h*_ and *C*_4*v*_ isomers, defined as the energy difference between the lowest-energy *D*_3*h*_ and *C*_4*v*_ structures of the Fe(CO)_5_^+^ cation and the lowest-energy structure of the neutral Fe(CO)_5_ molecule.

Table 1 lists the ionization energies (IE) for the *D*_3*h*_ and *C*_4*v*_ isomers of Fe(CO)_5_. VIE stands for the vertical IE defined as the energy difference between the cation in the geometry of the neutral molecule and the optimized geometry of the neutral molecule. IE_ad_(*D*_3*h*_) and IE_ad_(*C*_4*v*_) are the adiabatic IEs for *D*_3*h*_ and *C*_4*v*_ isomers, defined as the energy difference between the lowest-energy *D*_3*h*_ and *C*_4*v*_ structures of the Fe(CO)_5_^+^ cation and the lowest-energy structure of neutral Fe(CO)_5_. The adiabatic IEs obtained at the B3LYP/6-31+G(d) level of theory are the closest to the experimental ionization energies of Fe(CO)_5_, which vary from 7.95 to 8.6 eV according to the data compiled in the NIST Chemistry Webbook.^55^ Therefore, the B3LYP/6-31+G(d) method has been used in the subsequent calculations of potential energy scans for different covalent bonds and angles of Fe(CO)_5_^+^ to determine parameters of the rCHARMM force field.

The stability of the neutral (*D*_3*h*_) and cation (*C*_4*v*_) systems shown in Figure 1 was verified through the vibrational analysis calculated at the chosen B3LYP/6-31+G(d) level of theory, which indicated that all the vibrational frequencies in both systems were positive.

Table 2 lists covalent bonded (equilibrium bond lengths, force constants, and dissociation energies) and angular (equilibrium angles and force constants) interaction parameters for different Fe–C and C–O bonds in *D*_3*h*_ and *C*_4*v*_ isomers of Fe(CO)_5_^+^, calculated using the B3LYP/6-31+G(d) method. Atomic partial charges for singly charged and neutral Fe(CO)_5_, employed in the reactive MD simulations, were obtained through the natural bond orbital analysis using the Gaussian 16 software.^54^

**Table 2:** Parameters of Covalent Bonded and Angular Interactions, Eqs 1 and 2, for *D*_3*h*_ and *C*_4*v*_ Isomers of Fe(CO)_5_^+^ Employed in This Study

bond type	*r*_0_ (Å)	*k*_*ij*_^*r*^ (kcal/mol Å^–2^)	*D*_*ij*_ (kcal/mol)	*D*_*ij*_ (eV)
Fe(CO)_5_^+^ (*D*_3*h*_)				
Fe–C_ax_	1.89	122.9	39.9	1.73
Fe–C_eq_	1.90	99.2	29.7	1.29
C_ax/eq_–O	1.13	1494.3	223.5	9.69
Fe(CO)_5_^+^ (*C*_4*v*_)				
Fe–C_⊥_	1.94	85.7	26.4	1.15
Fe–C_∥_	1.90	116.1	36.0	1.56
C_⊥/∥_–O	1.13	1548.3	256.1	11.11

angle type	θ_0_ (deg)	*k*_*ijk*_^θ^ (kcal/mol rad^–2^)
Fe(CO)_5_^+^ (*D*_3*h*_)		
C_ax_–Fe–C_ax_	180	76.4
C_eq_–Fe–C_eq_	120	76.4
C_ax_–Fe–C_eq_	90	76.4
Fe–C_ax/eq_–O	180	28.0
Fe(CO)_5_^+^ (*C*_4*v*_)		
C_⊥_–Fe–C_∥_	96.4	100.0
C_∥_–Fe–C_∥_	89.3	100.0
Fe–C_⊥/∥_–O	180	28.0

Nonbonded van der Waals interactions between atoms of the system have been described by means of the Lennard-Jones potential:
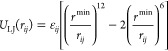
5where  and *r*^min^ = (*r*_*i*_^min^ + *r*_*j*_^min^)/2. The corresponding parameters are listed in Table 3.

**Table 3:** Parameters of the Lennard-Jones Potential, Eq 5, Describing the van der Waals Interaction between Atoms of Fe(CO)_5_^+^ and Argon Atoms

atom	ε (eV)	*r*^min^/2 (Å)	ref
Fe	0.0024	2.27	(56)
C	0.0041	1.95	(56)
O	0.0042	1.76	(56)
Ar	0.0103	1.91	(57)

### 2.2 Fragmentation of Isolated Fe(CO)_5_^+^

Simulations of electron-impact-induced fragmentation of an isolated iron pentacarbonyl cation have followed the methodology from ref (47). In the cited study, a model for irradiation-induced molecular fragmentation was developed on the basis of reactive MD simulations of W(CO)_6_^+^ fragmentation. Two scenarios of energy deposition into the target are considered within the model. (i) The localized energy deposition into a specific covalent bond immediately after the ionization or electron attachment processes. These processes happen on a sub-femtosecond scale and leave the molecular system in an excited electronic state. An excitation involving an antibonding molecular orbital evolves through the cleavage of a specific bond on the femtosecond time scale. (ii) Energy transfer into the system’s vibrational degrees of freedom via the electron–phonon coupling mechanism.^58^ This process happens on a picosecond time scale after the collision, and the subsequent molecular fragmentation may last up to microseconds.

Within the framework of classical reactive MD, we have simulated both the cleavage of individual covalent bonds and energy redistribution over all the molecular degrees of freedom. Both processes result in an increase in the cation’s internal energy after the energy deposition. The internal energy increase is treated as an initial increase in the kinetic energy of atoms. For simulations of the first fragmentation mechanism, the amount of energy *E* remaining in the system after ionization (i.e., excess energy over the first ionization potential) has been deposited locally into a specific covalent bond of the target and converted into the kinetic energy of the two atoms forming the bond. Velocities of these atoms have been defined to obey the total energy and momentum conservation laws:
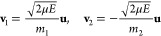
6Here *m*_1_, *m*_2_, and μ = *m*_1_*m*_2_/(*m*_1_ + *m*_2_) are respectively masses and the reduced mass of the atoms forming the bond, and **u** is a unit vector defining the direction of the relative velocity of these atoms upon bond cleavage.

The thermal mechanism of fragmentation corresponds to a statistical distribution of the deposited energy over all the degrees of freedom of the target. In this case, equilibrium velocities of atoms corresponding to a given temperature, *v*_*i*_^eq^, have been scaled by a factor α depending on the amount of deposited energy. The kinetic energy of *N* atoms is then given by
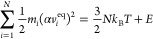
7The first term on the right-hand side of eq 7 is the kinetic energy of the atoms at the equilibrium temperature *T*, with *k*_B_ being the Boltzmann constant. The second term on the right-hand side is the excess energy deposited in the molecule during the collision.

The simulations of electron-impact-induced fragmentation of isolated Fe(CO)_5_^+^ have been performed based on the following computational protocol. First, the geometry of the Fe(CO)_5_^+^ cation was optimized by means of MBN Explorer using the parameters listed in Tables 2 and 3. Then, the cation was thermalized at *T* = 300 K; ten independent MD simulations of 1 ns duration each were performed. The simulations were performed using the Langevin thermostat with a damping time of 0.2 ps. In each simulated trajectory, atomic coordinates and velocities were recorded every 100 ps. The trajectories were used to generate a series of initial geometries and velocity distributions for the simulation of the fragmentation process.

The reactive MD simulations of Fe(CO)_5_^+^ fragmentation have been performed over 100 ns in a large simulation box with a side length of 200 Å. The simulations used the integration time step of 0.1 fs, and no thermostat was employed. 4000 constant-energy simulations were conducted for different values of the excess energy *E* ranging from 0 to ∼15.2 eV. In the case of energy deposition into specific covalent bonds of Fe(CO)_5_^+^, 30 to 70 independent runs for each value of *E* have been performed. For the simulations of the thermal mechanism of fragmentation, 30 runs for each value of excess energy were performed. The largest value of *E* considered here is about 10 times larger than the dissociation energy for a Fe–C bond (see Table 2), which enables the simulation of multiple Fe–C bond breaks. The amount of energy *E* has been varied from 0 to ∼10.8 eV in steps of ∼0.55 eV. At higher *E* values, a larger increment of ∼1.1 eV was considered. Molecular fragments produced at the end of 100 ns long simulations were analyzed. The corresponding fragment appearance energies were evaluated from this analysis and compared with experimental data.^20^

### 2.3 Fragmentation of Fe(CO)_5_^+^ Embedded into an Argon Cluster

The simulations of electron-impact-induced fragmentation of Fe(CO)_5_^+^ embedded into an argon cluster have been set up according to the experimental parameters from ref (24). In the cited study, the mixed Fe(CO)_5_@Ar compounds were prepared by passing the argon cluster beam via a pick-up cell filled with the vapor of iron pentacarbonyl; the resulting heterogeneous clusters were ionized by the electron impact.

The process of Fe(CO)_5_^+^ pick-up by argon clusters has been simulated by means of classical MD. First, a spherical argon cluster with a radius of 1.3 nm, containing 230 atoms, has been created using the modeler plug-in of MBN Studio.^42^ The cluster has been thermalized at 40 K over 1 ns. The interaction between argon atoms has been described using the Lennard-Jones potential, eq 5, with the parameters listed in Table 3. The simulations of Fe(CO)_5_^+^ pick-up on argon have been set up according to the experimental conditions of ref (24). A single Fe(CO)_5_^+^ thermalized at 300 K collided with a cold argon cluster thermalized at 40 K. The collision velocity was set equal to 4.9 Å/ps (490 m/s), corresponding to an average collision velocity in the experiment.^24^ The simulations have been performed for 10 ns.

The resulting geometry of a heterogeneous Fe(CO)_5_^+^@Ar cluster has been used as an input for the simulation of fragmentation of Fe(CO)_5_^+^ embedded in the cluster. The simulation protocol is similar to that described above in Section 2.2. An amount of energy *E* ranging from 0 to ∼21.7 eV was deposited into different Fe–C and C–O bonds of the cation. We have considered an increment of ∼2.2 eV over the whole energy range considered. In addition, the parameter *E* was varied in steps of ∼0.4 eV in the range *E* ≈ 4.3–8.7 eV. The selected energy range corresponds to the range of appearance energies reported in the experiment.^24^ The chosen increment of ∼0.4 eV corresponds to the experimental resolution reported in the cited study. 2800 MD simulations employing the rCHARMM force field have been carried out. The duration of each simulation was set to 25 ns with a time step of 0.1 fs.

## 3 Results and Discussion

### 3.1 Fragmentation of Isolated Iron Pentacarbonyl

**Figure 2:**
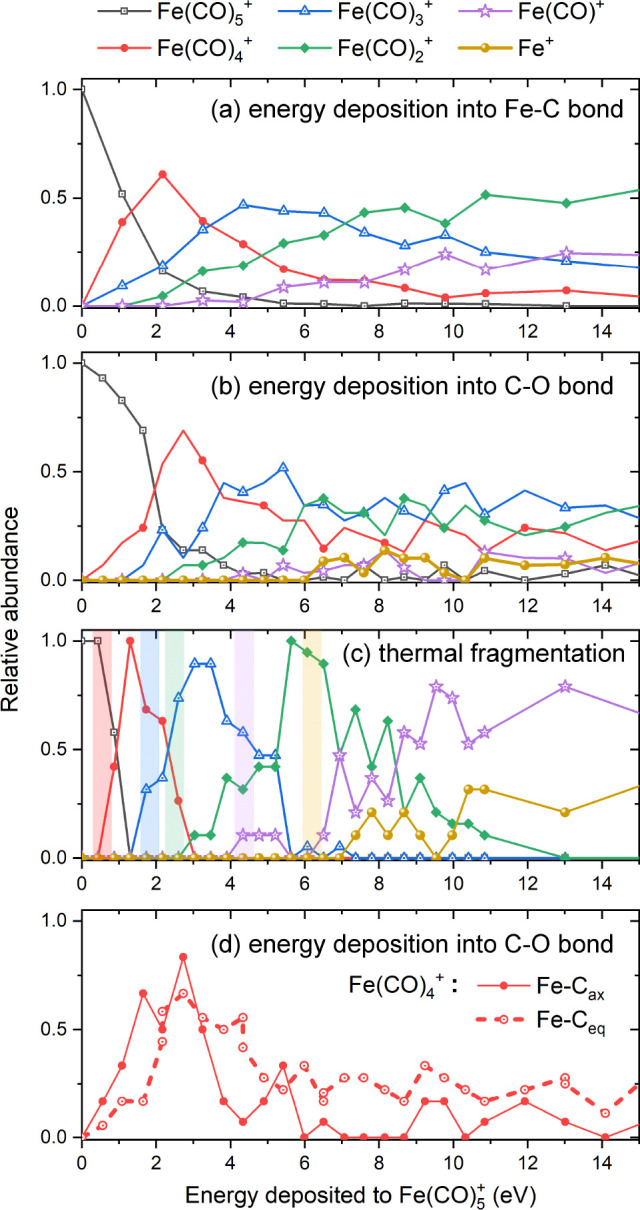
(a–c) Relative abundance of Fe(CO)_5–*n*_^+^ (*n* = 0–5) fragments produced after the electron-impact-induced fragmentation of isolated *D*_3*h*_ isomer of Fe(CO)_5_^+^ as a function of energy *E* deposited to the cation. Panels a and b describe the case of the localized energy deposition into a Fe–C bond and a C–O bond, respectively. Panel c describes the thermal mechanism of fragmentation where the energy is distributed over all degrees of freedom of the cation. Vertical color bars in panel c indicate the experimental appearance energies of Fe(CO)_5–*n*_^+^ (*n* = 1–5) fragments reported by Lacko et al.;^20^ the width of these bars corresponds to the experimental uncertainty. Legend corresponds to the data plotted in panels a, b, and c. (d) Relative abundances of Fe(CO)_4_^+^ fragments produced by the removal of one CO ligand. The solid line corresponds to the localized energy deposition in a Fe–C_ax_ coordinated C–O bond; the dashed line describes the case of energy deposition in a Fe–C_eq_ coordinated C–O bond.

**Figure 3:**
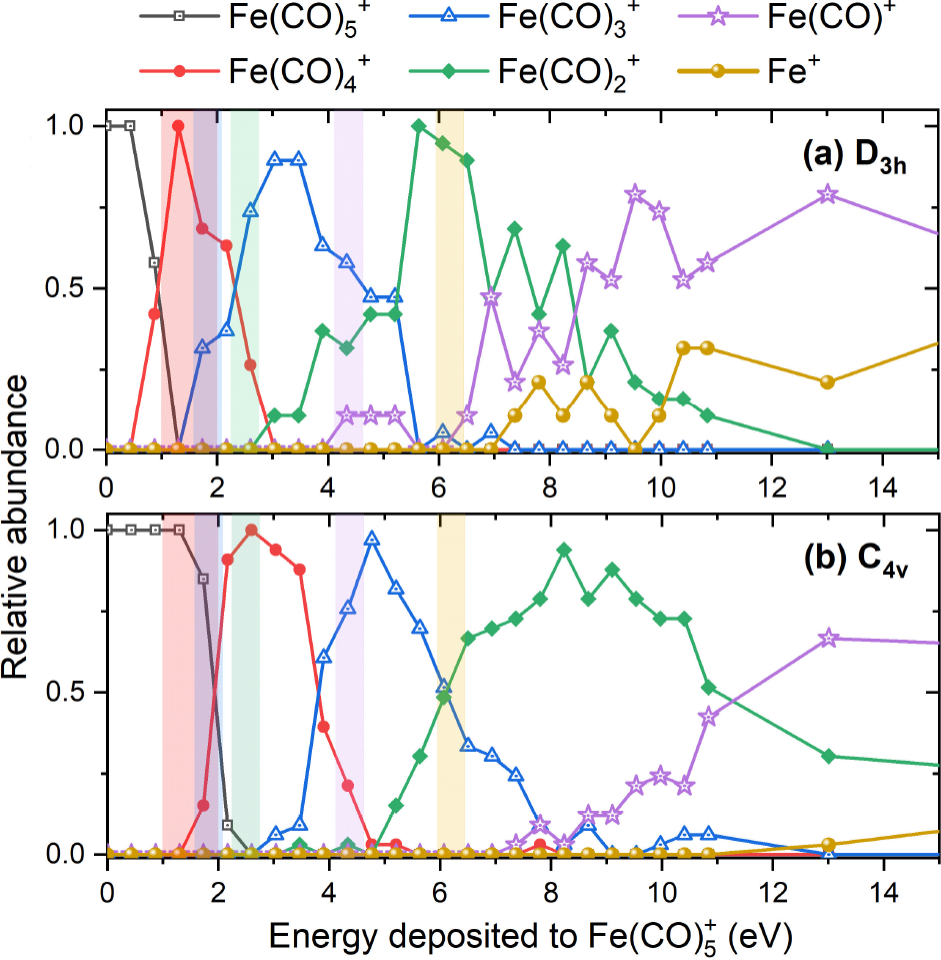
Relative abundance of Fe(CO)_5–*n*_^+^ (*n* = 0–5) fragments produced after the electron-impact-induced fragmentation of (a) *D*_3*h*_ and (b) *C*_4*v*_ isomers of isolated Fe(CO)_5_^+^ as a function of energy *E* deposited to the cation as a result of the collision. The presented distributions correspond to the thermal mechanism of fragmentation where the energy is distributed over all degrees of freedom. Vertical color bars indicate the experimental appearance energies of Fe(CO)_5–*n*_^+^ (*n* = 1–5) fragments reported by Lacko et al.;^20^ the width of these bars corresponds to the experimental uncertainty.

The main outcome of the performed simulations is the fragmentation patterns, that is, the abundance of different Fe(CO)_5–*n*_^+^ (*n* = 0–5) ionic products as a function of energy *E* deposited to the parent Fe(CO)_5_^+^ ion. These characteristics are plotted in Figures 2 and 3. As discussed in Section 2, we have considered the *D*_3*h*_ and *C*_4*v*_ structural isomers of Fe(CO)_5_^+^. While the former is similar to the structure of the neutral Fe(CO)_5_ molecule and is thus accessible upon vertical ionization, the latter requires considerable structural rearrangement. We have therefore assumed that in the case of the *D*_3*h*_ isomer it makes physical sense to distribute the excess energy initially into specific bonds, while the only realistic scenario for the *C*_4*v*_ structural isomer is the thermal distribution of energy.

We start the discussion with the nearly vertical ionization into the *D*_3*h*_ isomer of Fe(CO)_5_^+^. Figure 2a–c shows abundances of fragment ions for the cases when the excess energy has been deposited locally into one of Fe–C bonds or one of C–O bonds (panels a and b, respectively) and when the energy has been redistributed over all the degrees of freedom of the cation (panel c).

The abundance distributions for fragments produced after the localized energy deposition and the energy redistributed over all the molecular degrees of freedom have common features. In particular, each fragment ion has specific appearance energy; at larger values of the excess energy *E*, the abundance distribution reaches a maximum and then decreases with a further increase of *E*. The decrease is caused by the fact that if the ion is too “hot” a larger number of CO ligands are evaporated. Note that the horizontal axis in Figure 2, the energy deposited to Fe(CO)_5_^+^, can be converted into the energy of projectile electrons by adding the ionization energy of a Fe(CO)_5_ molecule, *I* = 8.45 eV, to this value. The vertical color bars in Figure 2c illustrate the appearance energies of Fe(CO)_5–*n*_^+^ (*n* = 1–5) fragments determined in mass-spectroscopic experiments by Lacko et al.,^20^ which have been converted into the excess energy deposited to Fe(CO)_5_^+^. Figure 2c indicates a very good agreement of the fragment appearance energies evaluated from the present simulations and the corresponding experimental values.

The qualitative similarity of the fragmentation patterns as a result of the different scenarios of energy deposition into the target cation points out to strong intramolecular vibrational redistribution (IVR). When the energy has been deposited to a specific bond (either Fe–C or C–O), it is transferred to the neighboring atoms and redistributed among the vibrational degrees of freedom. Thus, the subsequent dissociation dynamics proceeds similarly to the case when the energy has been distributed over all the degrees of freedom at the beginning of the simulation (see Figure 2c).

Still, there are several quantitative differences between the simulated fragmentation patterns. First, the lowest fragment appearance energies correspond to the localized energy deposition into a Fe–C bond (Figure 2a), followed by the case when the energy is deposited into a C–O bond (Figure 2b), and the highest appearance energies correspond to the thermal mechanism of fragmentation where the energy is distributed over all degrees of freedom of the cation (Figure 2c). The second difference concerns the width of the fragment yield curves as functions of the excess energy. The narrowest distributions correspond to the case of thermal fragmentation; somewhat broader distributions result from the energy deposited into a C–O bond, and the broadest distributions arise when the energy is deposited to a Fe–C bond.

As detailed in Section 2.2, dissociation energies for the Fe_ax_–C and Fe_eq_–C bonds differ by 0.44 eV (see Table 2). We have explored whether the resulting fragmentation pattern depends on the localized energy deposition to the C–O bonds coordinated to the different sites. The only detectable difference concerns the formation of the Fe(CO)_4_^+^ fragment, i.e., the loss of one ligand (see Figure 2d). Abundances of other fragments are very similar for the two considered cases. The efficient IVR leads to energy distribution over the entire cation and to the fact that the energy deposition into a C–O bond coordinated either to a C_ax_ site or a C_eq_ site (where it is bound much more weakly; see Table 2) plays a minor role in the dissociation process.

Figure 3 compares the fragmentation patterns for the *D*_3*h*_ and *C*_4*v*_ isomers of Fe(CO)_5_^+^ for the case when the excess energy has been thermally distributed over all the degrees of freedom of the cation. As discussed above, this is the only plausible distribution of excess energy for the structurally different *C*_4*v*_ isomer. Interestingly, the onsets of individual fragmentation channels are shifted to higher values for the *C*_4*v*_ isomer. The dissociation energies of different Fe–C bonds do not differ much between the two isomers (*D*_e_ = 1.29 and 1.73 eV in *D*_3*h*_ vs 1.15 and 1.56 eV in *C*_4*v*_; see Table 2). What is different is how the weaker and stronger Fe–C bonds are distributed within each isomer. In *D*_3*h*_, there are two stronger Fe–C_ax_ bonds (*D*_e_ = 1.73 eV) and three weaker Fe–C_eq_ bonds (*D*_e_ = 1.29 eV). In *C*_4*v*_, the perpendicular Fe–C_⊥_ bond is weaker (*D*_e_ = 1.15 eV) than the four Fe–C_∥_ bonds lying in a plane (*D*_e_ = 1.56 eV). The simulations show that the weak Fe–C_⊥_ bond in the *C*_4*v*_ isomer breaks first at deposition energies just below 2 eV, producing an almost planar and highly symmetric Fe(CO)_4_^+^ fragment, which is harder to break apart further.

This seemingly leads to a worse agreement with the experimental appearance energies for the *C*_4*v*_ isomer (see Figure 3b). However, two points should be noted here. First, the *C*_4*v*_ isomer is energetically lower by approximately 0.2 eV than the *D*_3*h*_ isomer (see Table 1). We did not account for this fact in the results plotted in Figure 3. Indeed, in both panels of Figure 3, the experimental appearance energies (which are measured with respect to the neutral ground state) were shifted by the same value of 8.45 eV; that is the most recent^20^ experimental value of the Fe(CO)_5_ ionization energy. Second, the spread of the literature IE values is relatively large (from 7.8 to 8.6 eV according to the data compiled in ref (55)), and a different choice of IE would change the position of vertical bars in Figure 3. Still, the much better agreement of the appearance energies of the *D*_3*h*_ isomer with the recent experimental values suggests that upon ionization the rearrangement of the cation to the energetically slightly stable isomer has a low probability.

It should be noted that the appearance energies of fragments are, in principle, the only quantities that can be directly compared to the mass-spectroscopic experimental data. In the electron impact ionization process
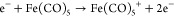
8the two outgoing electrons carry away a certain fraction of the incident electron energy. The excess energy stored in Fe(CO)_5_^+^ after the collision (the parameter which we control in the simulations) depends on the kinetic energy of these electrons. An (*e*, 2*e*) type of experiment, where the energies of all involved electrons are monitored in coincidence with the ionic fragmentation pattern,^59^ would be perfect for the comparison with the outcomes of present simulations. Unfortunately, we are not aware of any published data for (*e*, 2*e*) experiments with iron pentacarbonyl.

### 3.2 Fragmentation of Iron Pentacarbonyl Embedded into an Argon Cluster

In the experiments described in ref (24), iron pentacarbonyl molecules were picked up by an argon cluster beam. A pertinent question in such pick-up experiments is the structure of the resulting heterogeneous Fe(CO)_5_@Ar system;^60−63^ i.e., do Fe(CO)_5_ molecules stay on the surface of the cluster, or do they penetrate it and are embedded inside? This information is important for determining the further reactivity of the picked-up species and their interaction with incident electrons, which will depend on whether the molecules are covered by rare gas atoms or located on a cluster surface. This information cannot be obtained directly in experiments.

To address this question, we have simulated the collision of neutral and singly charged Fe(CO)_5_ molecules with an Ar_230_ cluster. The simulations of Fe(CO)_5_ pick-up on argon have been set up according to the experimental conditions of ref (24). As described above, a Fe(CO)_5_ molecule collided with an argon cluster with the velocity of 490 m/s, corresponding to an average collision velocity in the experiment.^24^ Three different collision geometries have been considered: (i) a central hit corresponding to the zero impact parameter, *b* = 0, (ii) a “lateral” hit with the impact parameter smaller than the radius of the cluster, *b* < *R*, and (iii) an “orbital” hit with *b* ∼ *R*. By considering different collision geometries, we have explored whether the molecule stays on top of the cluster or penetrates its interior region after the collision. Collision-induced evaporation of some loosely bound argon atoms has been observed in the performed simulations. As a result, after the collision, the Fe(CO)_5_^+^@Ar compound contained about 200 argon atoms.

**Figure 4:**
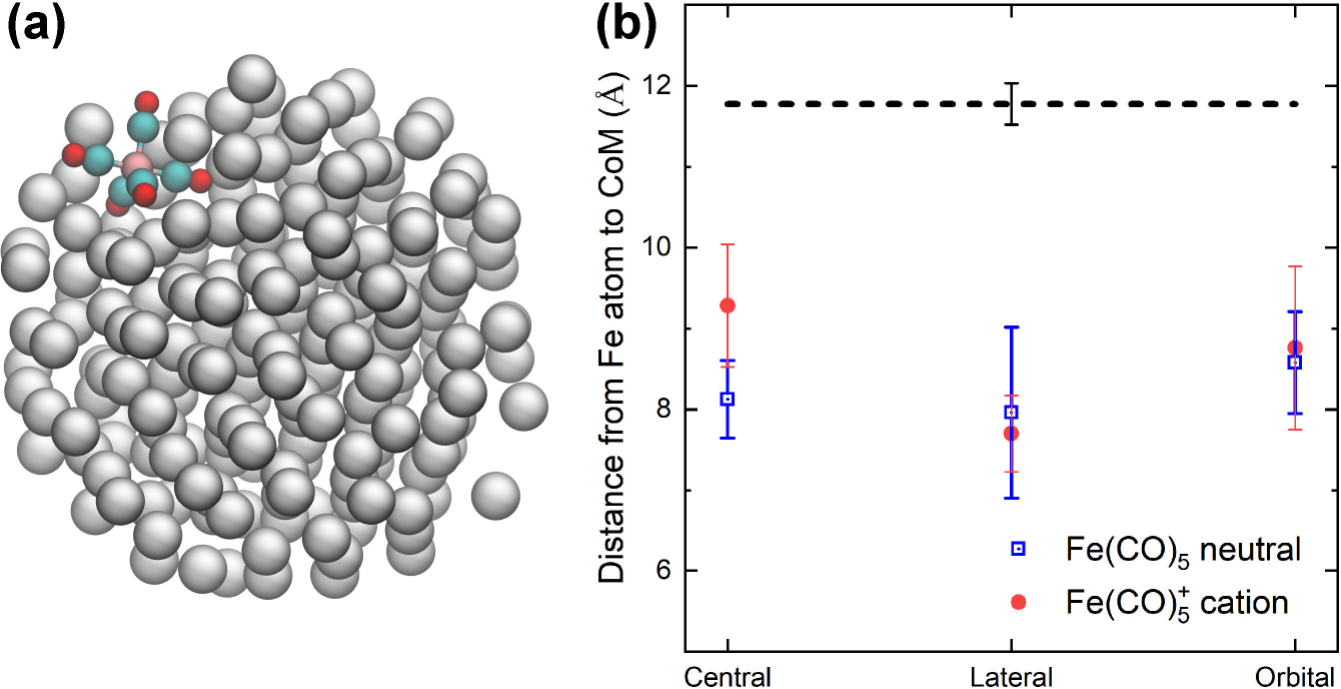
(a) An exemplary snapshot of the Fe(CO)_5_@Ar cluster formed after the collision of a Fe(CO)_5_ molecule with the argon cluster. (b) Average distance between the iron atom of Fe(CO)_5_ and the center of mass (CoM) of the cluster. The dashed line corresponds to the average radius of the Fe(CO)_5_@Ar cluster at the end of 10 ns long simulations. Five independent MD simulations of Fe(CO)_5_–Ar collisions have been performed for each charge state of Fe(CO)_5_ and each collision geometry; error bars indicate the corresponding standard deviation.

Figure 4a shows a typical structure of the Fe(CO)_5_@Ar cluster at the end of a 10 ns long simulation of the molecule pick-up process. Five independent trajectories have been simulated for each geometry of the molecule–cluster collision; the results of this analysis are shown in Figure 4b. The figure shows that the Fe(CO)_5_ molecule is embedded into the cluster but stays relatively close to the cluster surface. The average distance between the iron atom and the cluster surface varies between 3 and 4 Å, which is comparable with the distance between the Fe and O atoms in the Fe(CO)_5_^+^ cation (see Table 2).

The specific pick-up process in the above-described experiment proceeds via the argon cluster collision with a neutral Fe(CO)_5_ molecule. In this study, complementary simulations have been performed to study the pick-up of a singly charged Fe(CO)_5_^+^ (*D*_3*h*_) on the cluster at the same collision parameters. Such information can be useful e.g. for experiments using ions in argon matrices.^64^ As shown in Figure 4b, there is a minor difference in penetration of the neutral and ionic iron pentacarbonyl into the argon cluster, and the molecule’s charge state has a minor impact on the average distance between the iron atom and the center of mass of the cluster. A more detailed and systematic analysis of the geometry of Fe(CO)_5_^+^ inside argon clusters as a function of collision parameters goes beyond the scope of this study.

**Figure 5:**
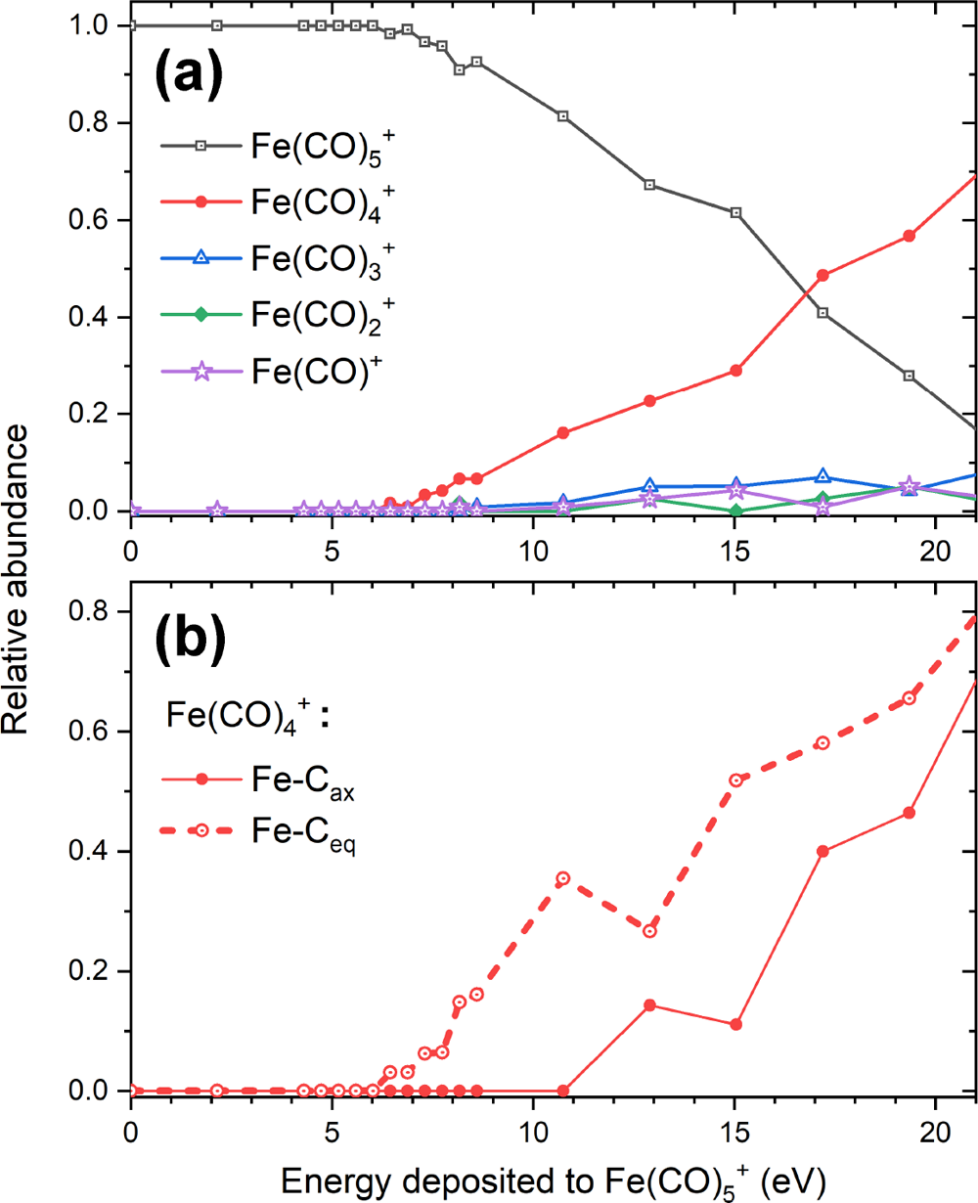
(a) Relative abundance of Fe(CO)_5–*n*_^+^ (*n* = 0–4) ionic species produced due to the electron-impact-induced fragmentation of Fe(CO)_5_^+^ embedded into the argon cluster. The excess energy was deposited locally into one of the C–O bonds. (b) Relative abundance of Fe(CO)_4_^+^ fragments. The solid line corresponds to the localized energy deposition in an axial (Fe–C_ax_ coordinated) C–O bond; the dashed line describes the case of energy deposition in an equatorial (Fe–C_eq_ coordinated) C–O bond.

Figure 5a shows the calculated relative abundances of Fe(CO)_5–*n*_^+^ (*n* = 0–4) ionic species produced due to the dissociation of Fe(CO)_5_^+^ embedded into the argon cluster. Only the *D*_3*h*_ isomer has been considered here for the following reasons. First, gas phase simulation results for this isomer agree better with the experimental data (see Figure 3). Second, considering the experimental procedure (pick-up of the neutral Fe(CO)_5_ molecule (*D*_3*h*_ isomer) into an argon cluster and subsequent ionization), we consider the structural rearrangement of Fe(CO)_5_^+^ to the *C*_4*v*_ isomer inside the argon cluster improbable.

The results shown in Figure 5a differ significantly from those obtained in the gas phase (see Figure 2). In the case of Fe(CO)_5_^+^@Ar, most of the Fe(CO)_5_^+^ ions have remained intact at the end of 25 ns long simulations (black curve with open squares), and the parent Fe(CO)_5_^+^ ion dominates the spectrum up to the deposited energies of *E* ∼ 17 eV. The only ionic fragment with non-negligible abundance is Fe(CO)_4_^+^ (red curve with circles), corresponding to the loss of one CO ligand. We note that in the simulations of Fe(CO)_5_^+^@Ar fragmentation all the excess energy has been deposited locally into one of the C–O bonds. The cause of the ligand stabilization is a fast energy transfer from atoms of a C–O bond to the argon environment, which serves as a heat bath that efficiently quenches the excess energy from the Fe(CO)_5_^+^ cation. Most dissociation events observed in the simulations have occurred on a time scale shorter than 100 ps, indicating a prompt fragmentation. The energy deposited into a C–O bond has been transferred into the vibrations of Fe–C bonds, eventually leading to the breakage of the metal–ligand bonds. This process occurs before the energy is quenched by the argon environment.

The observed fragmentation pattern has an interesting consequence. Figure 5b shows the Fe(CO)_4_^+^ abundance for the case of the localized energy deposition into the axial or equatorial C–O bonds. The appearance energies for the Fe(CO)_4_^+^ fragment differ in these two cases by more than 4 eV. Therefore, we conclude that the difference in the strength of the metal–ligand bonds (∼0.5 eV) has a strong impact on the efficiency of the energy transfer to the argon environment. This result is in strong contrast to the case of an isolated cation, where the dissociation was not prompt but thermally driven, and the difference between the two types of bonds was much smaller (see Figure 2d).

An important observation from ref (24) should also be mentioned here in connection to the present simulation outcomes. The measurements of appearance energies revealed that a substantial fraction of Fe(CO)_5_ molecules on the cluster are not ionized by direct electron impact, but rather the electron ionizes argon atoms in the cluster, and Fe(CO)_5_ is then ionized by a hole transfer from argon.^24^ This was concluded based on the fact that the appearance energies of fragments coincided with the ionization energy of an argon atom. This experimental observation is supported by the results shown in Figure 4 that the Fe(CO)_5_ molecule is embedded into the cluster after the pick-up. Considering the ionization potentials of an argon atom (15.8 eV)^55^ and a Fe(CO)_5_ molecule (8.45 eV),^20^ such charge transfer is exothermic by ∼7.3 eV. As demonstrated in Figure 5a, almost all ions formed in this way remain intact and do not experience fragmentation.

**Figure 6:**
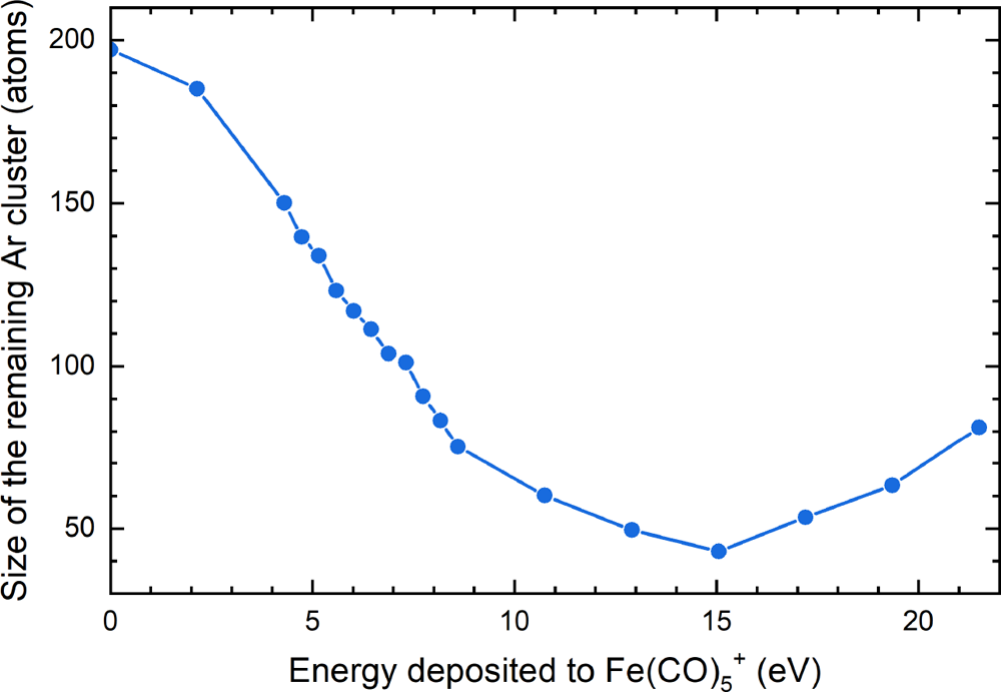
Average number of argon atoms in the cluster surrounding the Fe(CO)_5_^+^ ion at the end of the 25 ns long simulation as a function of energy deposited into Fe(CO)_5_^+^.

The energy transferred to the cluster is redistributed among its internal degrees of freedom, which leads to the evaporation of the weakly bound argon atoms. Figure 6 shows the remaining number of argon atoms in the cluster at the end of the 25 ns long simulations as a function of energy deposited into Fe(CO)_5_^+^, *E*. As the deposited energy increases, the resulting cluster size decreases, which is expected. However, this trend breaks at the deposited energy of *E* ∼ 15 eV. The main reason for this phenomenon is that for larger values *E*, a larger number of CO ligands are released by a prompt dissociation with a small energy loss to argon atoms, yielding mainly Fe(CO)_4_^+^ as demonstrated in Figure 5a.

## 4 Conclusions

In conclusion, the dissociative ionization (DI) of the iron pentacarbonyl molecule, Fe(CO)_5_, has been studied by means of reactive molecular dynamics simulations using the MBN Explorer software package.^34^ The main focus of this study concerned the quantitative analysis of different ionic fragments and their appearance energies for the fragmentation of single Fe(CO)_5_^+^ in the gas phase, and this cation embedded into a molecular environment.

For isolated Fe(CO)_5_^+^, two structural isomers were considered. The evaluated fragment appearance energies were in better agreement with experimental values^20^ for the *D*_3*h*_ isomer, even though it is by ∼0.2 eV less stable than the *C*_4*v*_ isomer. The main outcome of the simulations—abundances of individual fragments—was explored for the *D*_3*h*_ isomer and shows a surprisingly little dependence on the initial conditions. This observation is attributed to intramolecular vibrational redistribution (IVR), which means that on a short time scale the excess energy becomes distributed over the internal degrees of freedom of the whole cation, and the dissociation of metal–ligand bonds in Fe(CO)_5_^+^ proceeds via the thermal mechanism of fragmentation.

In the case of iron pentacarbonyl embedded into an argon cluster, the release of CO ligands is strongly suppressed. The simulation results reported in this study provide an atomistic understanding of the cluster-beam study of Lengyel et al.,^24^ who observed such stabilization experimentally. We have demonstrated that the excess energy deposited to the Fe(CO)_5_^+^ cation as a result of the electron collision is efficiently quenched by the argon environment, which leads to the heating of the cluster and the evaporation of weakly bound argon atoms. The simulations performed in this study also bring experimentally inaccessible information about the structure of the heterogeneous Fe(CO)_5_^+^@Ar cluster following the pick-up collision of Fe(CO)_5_ molecules with pristine argon clusters. It has been demonstrated that Fe(CO)_5_ is embedded into the argon cluster, and the penetration depth of the picked-up molecule does not depend on its charge state (a neutral or a singly charged positive species).

The present findings are relevant for understanding the irradiation-driven fragmentation of molecular systems placed in molecular environments. Several effects related to a molecular environment and influencing the fragmentation degree have been discussed in the literature, such as mechanical caging,^65^ stabilization of transient species,^66^ change of chemical pathways,^67^ or polarization effects.^25,68^ The present findings elucidate in a quantitative way one of the most common effects of the molecular environment—quenching of the excess energy deposited into the system during the fragmentation process.

The simulations performed in this study are also relevant to the question of the DI of precursor molecules during the focused electron beam-induced deposition (FEBID) process. Even though the relative contribution of DI (with respect to the dissociative electron attachment and neutral dissociation processes) varies with the energy of secondary electrons in a given deposition process, this contribution is always significant.^13,69^ The efficient IVR mechanism observed and analyzed in this study indicates that the iron pentacarbonyl cations are almost always vibrationally excited, and the loss of CO ligands is a thermally driven process. When the molecules are physisorbed on a substrate—as in the case of FEBID—the vibrational energy can be efficiently quenched by the environment. The process of quenching is similar to that observed here by the argon cluster. Understanding irradiation-driven fragmentation patterns for molecular systems in environments facilitates the advancement of advanced computational models for studying irradiation-induced chemistry processes involving complex molecular systems.^15,16,33,36^
